# Neurotoxicity, Neuroprotection, In Vitro MAOA/MAOB Inhibitory Activity Assessment, Molecular Docking, and Permeability Assay Studies of Newly Synthesized Hydrazones Containing a Pyrrole Ring

**DOI:** 10.3390/molecules29184338

**Published:** 2024-09-12

**Authors:** Maya Georgieva, Emilio Mateev, Iva Valkova, Hristina Kuteva, Diana Tzankova, Denitsa Stefanova, Yordan Yordanov, Karolina Lybomirova, Alexander Zlatkov, Virginia Tzankova, Magdalena Kondeva-Burdina

**Affiliations:** 1 Department Pharmaceutical Chemistry, Faculty of Pharmacy, Medical University of Sofia, 1000 Sofia, Bulgaria; e.mateev@pharmfac.mu-sofia.bg (E.M.); d.tsankova@pharmfac.mu-sofia.bg (D.T.); azlatkov@pharmfac.mu-sofia.bg (A.Z.); 2 Department Chemistry, Faculty of Pharmacy, Medical University of Sofia, 1000 Sofia, Bulgaria; ivalkova@pharmfac.mu-sofia.bg; 3 Department Pharmacology, Pharmacotherapy and Toxicology, Faculty of Pharmacy, Medical University of Sofia, 1000 Sofia, Bulgaria; hristina.kuteva@abv.bg (H.K.); denitsa.stefanova@pharmfac.mu-sofia.bg (D.S.); yyordanov@pharmfac.mu-sofia.bg (Y.Y.); vtzankova@pharmfac.mu-sofia.bg (V.T.); mkondeva@pharmfac.mu-sofia.bg (M.K.-B.); 4 Department Occupational Medicine, Faculty of Public Health, Medical University of Sofia, 1000 Sofia, Bulgaria; k.lybomirova@foz.mu-sofia.bg

**Keywords:** neurotoxicity, neuroprotection, *h*MAOA/*h*MAOB, pyrrole, hydrazones

## Abstract

Neurodegenerative diseases such as Parkinson’s and Alzheimer’s continue to be some of the most significant challenges in modern medicine. Recent research related to the molecular mechanisms of parkinsonism has opened up new approaches to antiparkinsonian therapy. In response to this, we present the evaluation of the potential neuroprotective and MAOA/MAOB inhibitory effects of newly synthesized hydrazones, containing a pyrrole moiety in the carboxyl fragment of the structure. The substances were studied on different brain subcellular fractions, including rat brain synaptosomes, mitochondria, and microsomes. The single application of 50 µM of each compound to the subcellular fractions showed that all substances exhibit a weak neurotoxic effect, with **7b**, **7d**, and **8d** being the least neurotoxic representatives. The corresponding neuroprotective and antioxidant effects were also evaluated in different injury models on subcellular fractions, single out **7b**, **7d**, and **8d** as the most prominent derivatives. A 1 µM concentration of each molecule from the series was also studied for potential *h*MAOA/*h*MAOB inhibitory effects. The results revealed a lack of *h*MAOA activity for all evaluated structures and the appearance of *h*MAOB effects, with compounds **7b**, **7d**, and **8d** showing effects similar to those of selegiline. The best *h*MAOB selectivity index (>204) was determined for **7d** and **8d**, distinguishing these two representatives as the most promising molecules for further studies as potential selective MAOB inhibitors. The performed molecular docking simulations defined the appearance of selective MAOB inhibitory effects based on the interaction of the tested molecules with Tyr398, which is one of the components of the aromatic cage of MAOB and participated in π–π stabilization with the aromatic pyrrole ring. The preliminary PAMPA testing indicated that in relation to the blood–brain barrier (BBB) permeability, the tested pyrrole-based hydrazones may be considered as high permeable, except for **8a** and **8e**, which were established to be permeable in the medium range with −logP of 5.268 and 5.714, respectively, compared to the applied references.

## 1. Introduction

Parkinson’s disease (PD) is a neurodegenerative disorder that develops relatively rapidly and is characterized by the death or damage of dopaminergic neurons in the *substantia nigra*. Pathophysiologically, it is manifested by the presence of so-called Lewy bodies and alpha-synuclein protein complexes. In addition to the dopamine-producing structures, the structures responsible for the production of serotonin, acetylcholine, and noradrenaline in different parts of the brain are also disrupted [1]. The progressive loss of dopaminergic neurons leads to a reduction in the amount of dopamine in the *substantia nigra* and the onset of the symptoms of the disease, which most commonly include tremor, rigidity, bradykinesia, and numbness [2]. The first and most common symptom is tremor, which is expressed by involuntary trembling at rest of the limbs and, in some cases, parts of the face [3]. Another distinctive symptom is rigidity, which is characterized by resistance to movement, most commonly described in the literature as “rigor” [4]. Bradykinesia is another symptom that is expressed in slow movements and is one of the main and most severe symptoms, as well as a cause of reduced quality of life in patients. It manifests itself due to the inability of the basal ganglia to properly conduct movement commands [5]. The progressive loss of neuronal function in this condition seriously affects patients’ lives, with conventional treatments often not offering satisfactory symptom relief or halting disease progression.

The most common enzyme regulating dopamine efficiency is monoamine oxidase (MAO). This enzyme exists in two isoforms—monoamine oxidase A (MAO-A) and monoamine oxidase B (MAO-B). MAO-A is mainly distributed in the gastrointestinal tract, platelets, heart, catecholaminergic neurons, such as dopaminergic neurons in substantia nigra (SN), norepinephrine neurons in locus coeruleus, etc. [6] and can promote the metabolism of tyramine, while MAO-B is mainly found in platelets and glial cells. This distribution determines the total MAO activity within the brain as defined by approximately 20% MAO-A and 80% MAO-B [7,8,9,10]. Both isoenzymes are closely related to the oxidative deamination of dopamine, phenethylamine, 5-hydroxytryptamine, and norepinephrine, known as basic monoamine neurotransmitters in the brain. MAO-A metabolizes dopamine in presynaptic neurons, while MAO-B metabolizes dopamine released to synaptic cleft and taken up by glial cells, thus affecting different regions in the brain.

**Figure 1:**
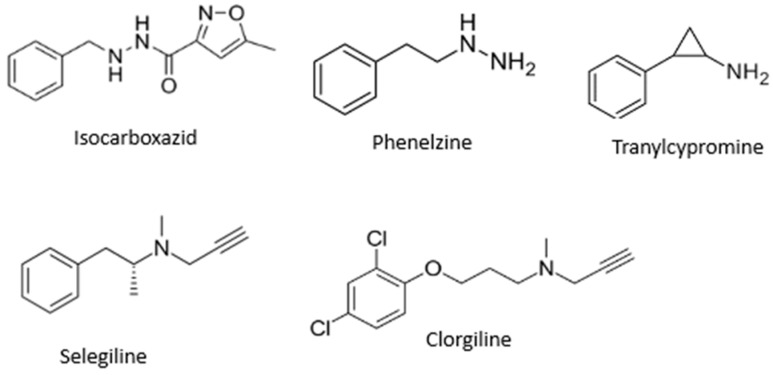
Structures of some MAO inhibitors.

MAO inhibitors indirectly affect dopaminergic neurotransmission and find application as agents for treatment of PD. MAO-B inhibitors inhibit MAO-B activity in the brain, block dopamine catabolism, enhance dopamine signaling, and selectively enhance dopamine levels at synaptic cleft [9], while MAO-A inhibitors are mainly applied as antidepressants [11]. The most known MAO inhibitors applied in therapy include, but are not limited to, isocarboxazid, phenelzine, tranylcypromine, selegiline, and clorgiline (Figure 1).

Pyrrole is a heterocyclic aromatic organic compound characterized by its role in many biologically significant structures. Its derivatives exhibit diverse pharmacological activities, including, but not limited to: anti-inflammatory [12], neuroprotective, antitumor [13,14,15], antidepressant [16], antiepileptic and anticonvulsant [12], antidiabetic [17], antitubercular [18,19,20,21,22], inhibitors of HMG-CoA reductase [23], antipsychotic [12], etc. Some pyrrole derivatives have been shown to exhibit marked antioxidant activity [24,25]. In addition, numerous studies have been conducted on the potential inhibitory activity of pyrrole derivatives on MAO enzyme activity [26,27].

The diverse pharmacological effects (including neurological) and their low toxicity make pyrrole derivatives interesting candidates for novel therapeutic agents in neurodegenerative diseases (including Parkinson’s disease).

## 2. Results

### 2.1. Chemistry

**Figure 2:**
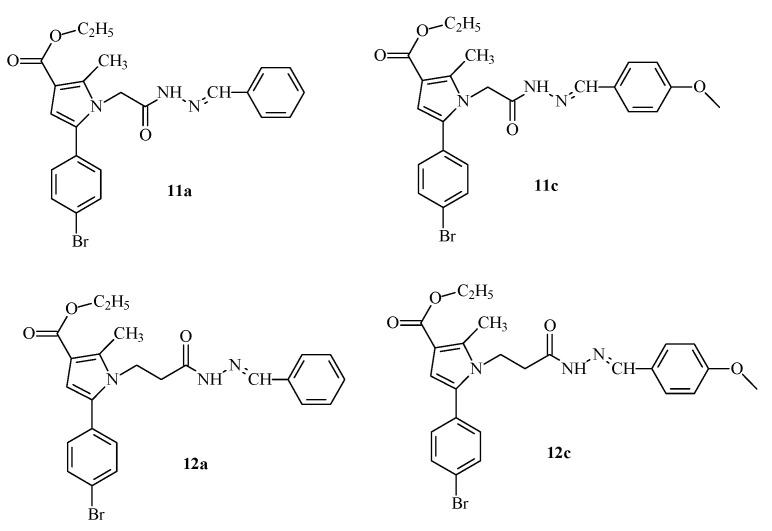
Structures of the most active molecule **12a** and its analogues containing a benzaldehyde residue from a previously synthesized series [28].

Some promising effects of previously synthesized from our group pyrrole-hydrazones obtained through condensation with a number of substituted benzaldehydes have been demonstrated and published [28] (Figure 2). 

**Figure 3:**
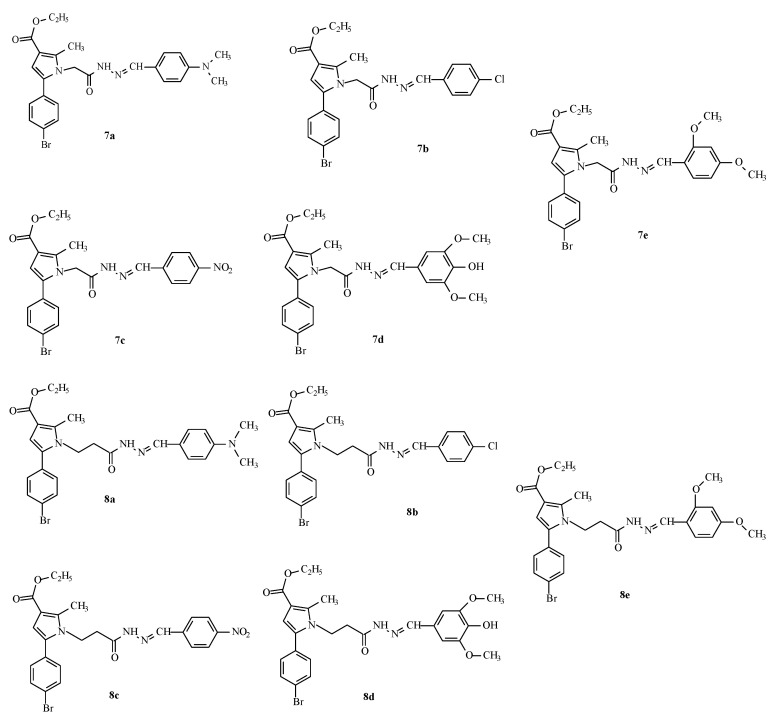
Structures and IDs of the evaluated hydrazones.

This encouraged us to enrich the series, attempting to optimize the activity of the identified as most active derivative **12a** by synthesis of some analogues through an optimized synthetic procedure identified in Tzankova et al., 2023 [29]. The structures of the target hydrazones are presented in Figure 3.

### 2.2. In Vitro Neurotoxicity Evaluation

The neurotoxicity of the tested hydrazones was evaluated on different subcellular fractions, aiming to identify the effect of the substances on the biomarkers characterizing the functional-metabolic status profile of the cellular and subcellular fractions.

#### 2.2.1. Effects of Newly Synthesized Hydrazones on Isolated Rat Brain Synaptosomes

**Figure 4:**
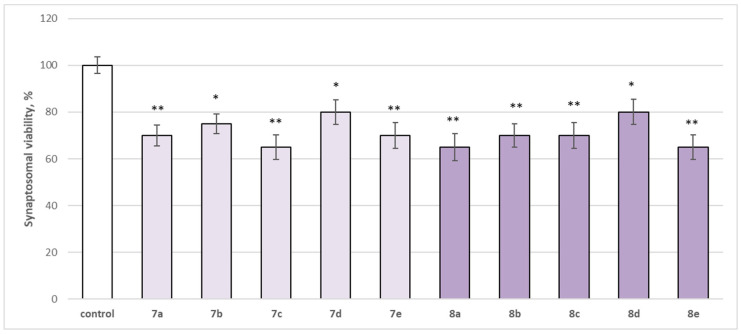
Effect of the test substances applied alone at a concentration of 50 µM on synaptosomal viability. * *p* < 0.05; ** *p* < 0.01 vs. control (non-treated synaptosomes).

**Figure 5:**
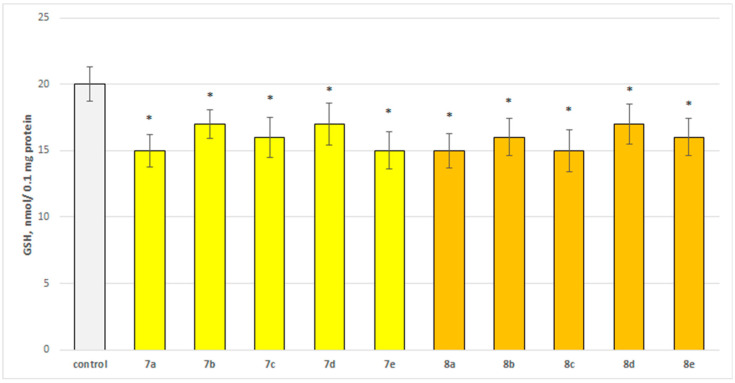
Effect of the test substances applied alone at a concentration of 50 µM on the level of glutathione (GSH). * *p* < 0.05 vs. control (non-treated synaptosomes).

The neurotoxicity of the target compounds on biomarkers, characterizing the functional-metabolic profile of brain synaptosomes, was evaluated. The results indicated the appearance of statistically significant weak neurotoxicity when compared to the control (untreated synaptosomes). The test compounds (**7a**–**7e** and **8a**–**8e**) were administered to brain synaptosomes alone at a concentration of 50 µM. The derivatives slightly decreased synaptosomal viability and reduced glutathione levels, as visualized in Figure 4 and Figure 5. The substances with the weakest neurotoxic effect were **7b**, **7d**, and **8d**.

Substances **7a**, **7e**, **8b**, and **8c** statistically significantly decreased synaptosomal viability by 30%; **7c**, **8a**, and **8e** decreased by 35%; **7b**—by 25%; and **7d** and **8d**—by 20% compared to the control.

Substances **7a**, **7e**, **8a**, and **8c** statistically significantly decreased the glutathione (GSH) level by 25%; **7c**, **8b**, and **8e** decreased by 20%; and **7b**, **7d**, and **8d** decreased by 15% compared to the control.

#### 2.2.2. Effects of Newly Synthesized Hydrazones on Isolated Rat Brain Mitochondria

**Figure 6:**
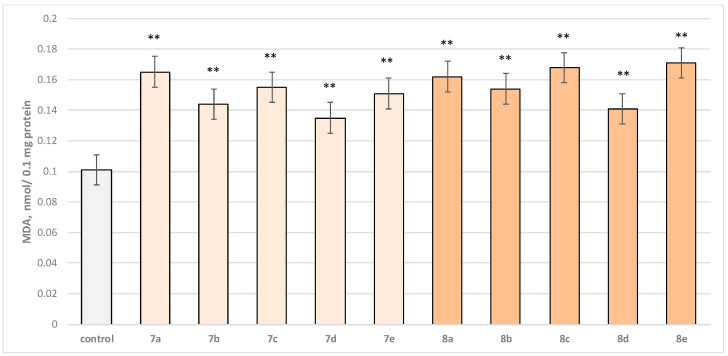
Effect of test substances applied alone at a concentration of 50 µM on MDA production. ** *p* < 0.01 vs. control (non-treated mitochondria).

**Figure 7:**
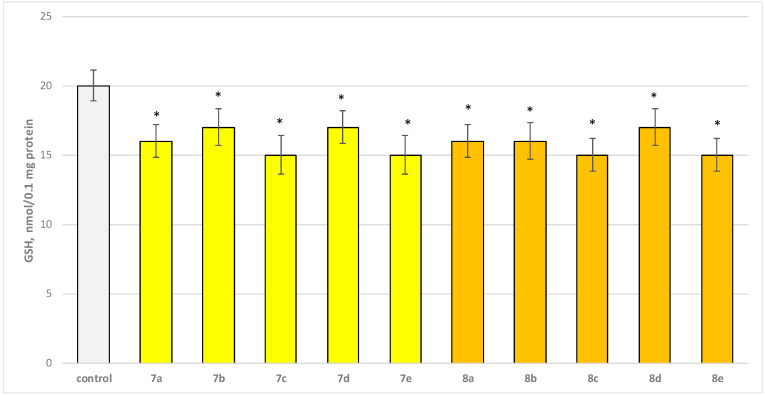
Effect of test substances applied alone at a concentration of 50 µM on GSH level. * *p* < 0.05 vs. control (non-treated mitochondria).

Two characteristic parameters determining the functional-metabolic profile of brain mitochondria were evaluated in this experimental procedure. The effects of the target molecules on malondialdehyde (MDA) production and GSH levels were followed, and it was found that when applied alone, at a concentration of 50 µM, to brain mitochondria, the test substances (**7a**–**7e** and **8a**–**8e** series) again exhibited a statistically significant weak neurotoxic effect, relative to the control (untreated mitochondria). A slight increase in malondialdehyde (MDA) production and a decrease in the reduced glutathione (GSH) level are observed as presented in Figure 6 and Figure 7. The substances with the weakest neurotoxic effect were **7b**, **7d**, and **8d**.

Substance **7a** increased MDA production statistically significantly by 63%; **7b** increased by 43%; **7c**—by 53%; **7d**—by 34%; **7e**—by 50%; **8a**—by 60%; **8b**—by 52%; **8c**—by 66%; **8d**—by 40%; and **8e**—by 69% compared to the control.

Substances **7a**, **8a**, and **8b** decreased GSH level statistically significantly by 20%; **7b**, **7d**, and **8d** decreased by 15%; **7c**, **7e**, **8c**, and **8e**—by 25% compared to the control.

#### 2.2.3. Effects of Newly Synthesized Hydrazones on Isolated Rat Brain Microsomes

**Figure 8:**
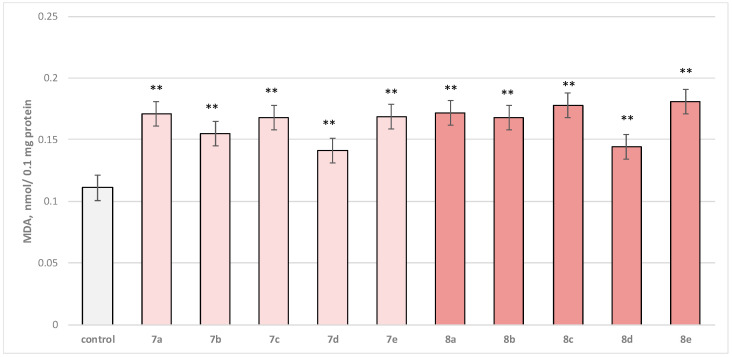
Effect of test substances applied alone, at a concentration of 50 µM, on MDA production. ** *p* < 0.01 vs. control (non-treated microsomes).

Self-administered, at a concentration of 50 µM, to brain microsomes, the test substances (series **7a**–**7e** and **8a**–**8e**) manifested a statistically significant weak neurotoxic effect relative to the control (untreated microsomes). In this in vitro model for neurotoxicity assessment, the production of malondialdehyde (MDA) was tracked as a marker of lipid peroxidation. A slight increase in this parameter was observed, with **7b**, **7d**, and **8d** outstanding as the weakest neurotoxic derivatives (Figure 8). 

Substance 7a increased MDA production statistically significantly by 54%; 7b increased by 40%; **7c**—by 51%; **7d**—by 27%; **7e**—by 52%; **8a**—by 55%; **8b**—by 51%; **8c**—by 60%; **8d**—by 30%; and **8e**—by 63%, compared to the control.

### 2.3. In Vitro Evaluation of Neuroprotective Effects of the Target Compounds

The neuroprotective properties of the tested hydrazones were evaluated on different subcellular fractions, applying different models for neurotoxicity induction.

#### 2.3.1. Effect of the Target Substances in a Model of 6-OHDA-Induced Neurotoxicity on Isolated Rat Brain Synaptosomes

**Figure 9:**
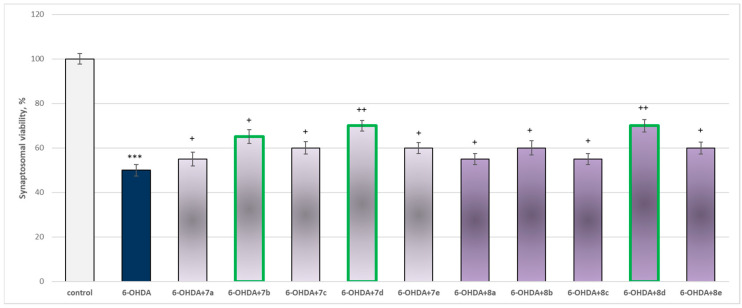
Effect of substances in combination with 6-OHDA on synaptosomal viability. *** *p* < 0.001 vs. control (non-treated synaptosomes); + *p* < 0.05; ++ *p* < 0.01 vs. 6-OHDA. The green color indicates the most active derivatives.

**Figure 10:**
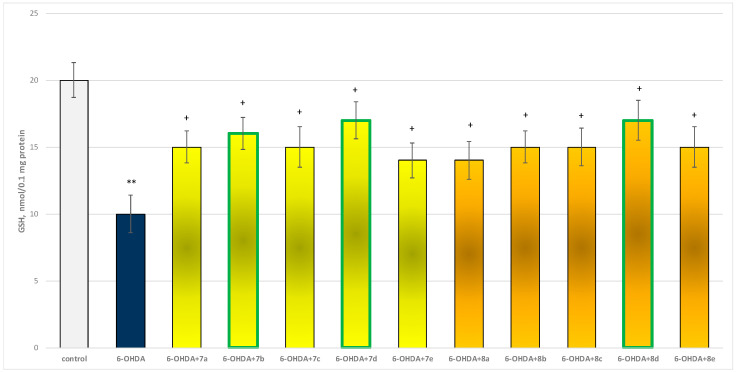
Effect of substances, in combination with 6-OHDA, on the level of reduced glutathione (GSH). ** *p* < 0.001 vs. control (non-treated synaptosomes); + *p* < 0.05 vs. 6-OHDA. The green color indicates the most active derivatives.

The toxic agent (6-OHDA), applied alone at a concentration of 150 µM, to isolated synaptosomes resulted in a statistically significant 50% decrease in synaptosomal viability and GSH level compared to the control (untreated synaptosomes) (Figure 9 and Figure 10).

When combined with 6-OHDA, all substances (at a concentration of 50 µM) exhibited a statistically significant neuroprotective effect against the toxic agent. However, the substances **7b**, **7d**, and **8d** showed the most pronounced neuroprotection on the studied parameters (Figure 9 and Figure 10).

Substances **7a**, **8a**, and **8c** statistically significantly preserved synaptosomal viability by 10%; **7c**, **8e**, **8b**, and **8e** preserved by 20%; **7b**—by 30%; and **7d** and **8d**—by 40%, compared to the toxic agent (6-OHDA).

Substances **7a**, **7c**, **8b**, **8c**, and **8e** statistically significantly preserved GSH levels by 50%; **7e** and **8a** preserved by 40%; **7b**—by 60%; and **7d** and **8d**—by 70%, compared to the toxic agent (6-OHDA).

#### 2.3.2. Effect of the Target Substances in a Model of *t*-BuOOH-Induced Oxidative Stress on Isolated Rat Brain Mitochondria

**Figure 11:**
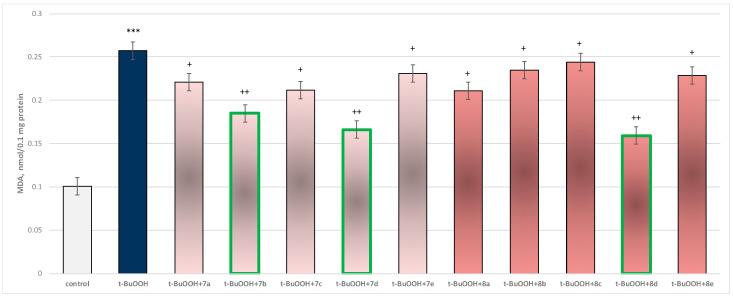
Effect of substances in combination with *t*-BuOOH on MDA production. *** *p* < 0.001 vs. control (non-treated mitochondria); + *p* < 0.05; ++ *p* < 0.01 vs. *t*-BuOOH. The green color indicates the most active derivatives.

**Figure 12:**
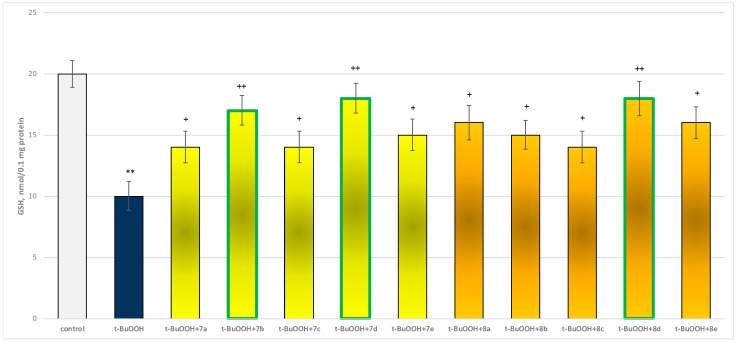
Effect of substances in combination with *t*-BuOOH on GSH level. ** *p* < 0.01 vs. control (non-treated mitochondria); + *p* < 0.05; ++ *p* < 0.01 vs. *t*-BuOOH. The green color indicates the most active derivatives.

In isolated brain mitochondria, *t*-BuOOH applied alone statistically significantly increased MDA production by 154% and decreased GSH level by 50%, compared to control (untreated mitochondria) (Figure 11 and Figure 12).

When combined with *t*-BuOOH, again all substances (at a concentration of 50 µM) exhibited a statistically significant neuroprotective effect compared to the toxic agent. The substances **7b**, **7d**, and **8d** again showed the most pronounced neuroprotection on the parameters studied (Figure 11 and Figure 12).

The protective effects are probably related on one hand to the storage of the reduced glutathione and, on the other, to the possible scavenging of free radicals produced by *t*-BuOOH metabolism.

Substance **7a** reduced MDA production statistically significantly by 14%; **7b** reduced by 28%; **7c**—by 18%; **7d**—by 35%; **7e**—by 10%; **8a**—by 18%; **8b**—by 9%; **8c**—by 5%; **8d**—by 38%; and **8e**—by 11%, compared to the toxic agent (*t*-BuOOH).

Substances **7a**, **7c**, and **8c** preserved GSH levels statistically significantly by 40%; **7b** preserved by 70%; **7d** and **8d**—by 80%; **7e** and **8b**—by 50%; and **8a** and **8e**—by 60%, compared to the toxic agent (*t*-BuOOH).

#### 2.3.3. Effect of the Target Substances in a Model of Fe^2+^/AA-Induced Oxidative Stress on Isolated Rat Brain Microsomes

**Figure 13:**
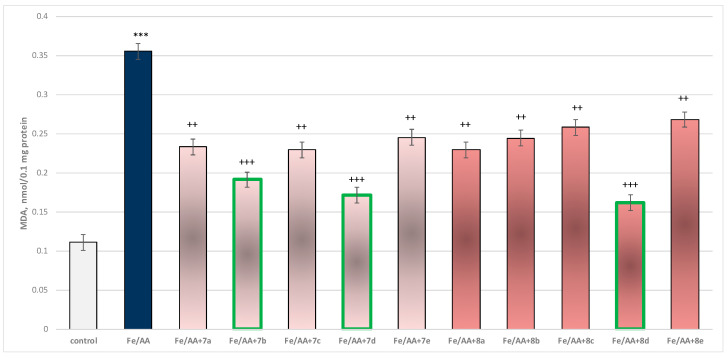
Effect of substances under conditions of non-enzyme-induced lipid peroxidation (Fe^2+^/AA). *** *p* < 0.001 vs. control (non-treated microsomes); ++ *p* < 0.01; +++ *p* < 0.001 vs. Fe^2+^/AA. The green color indicates the most active derivatives.

Incubation of brain microsomes with Fe^2+^/AA resulted in a statistically significant 220% increase in MDA production relative to control (untreated microsomes) (Figure 13).

In combination with Fe^2+^/AA, again all substances (at a concentration of 50 µM) exhibited a statistically significant antioxidant effect against the toxic agent. The substances **7b**, **7d**, and **8d** again had the most prominent effect on MDA production (Figure 13).

Substance **7a** decreased MDA production statistically significantly by 34%; **7b** decreased by 46%; **7c**—by 35%; **7d**—by 52%; **7e**—by 31%; **8a**—by 35%; **8b**—by 31%; **8c**—by 27%; **8d**—by 54%; and **8e**—by 25%, compared to the toxic agent (Fe^2+^/AA).

### 2.4. In Vitro Evaluation of the Enzymatic Activity of the Target Molecules on Human Recombinant MAOA/MAOB Enzyme

**Figure 14:**
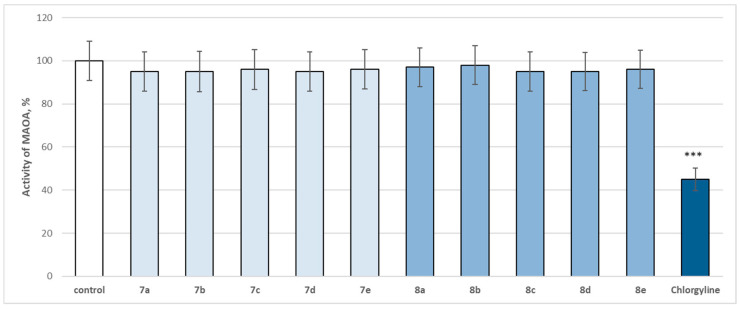
Effect of newly synthetized hydrazones containing a pyrrole cycle in the carboxyl fragment of the structure (at 1 µM concentration) on the activity of human recombinant MAOA enzyme (*h*MAOA). *** *p* < 0.001 vs. control (pure *h*MAOA).

**Figure 15:**
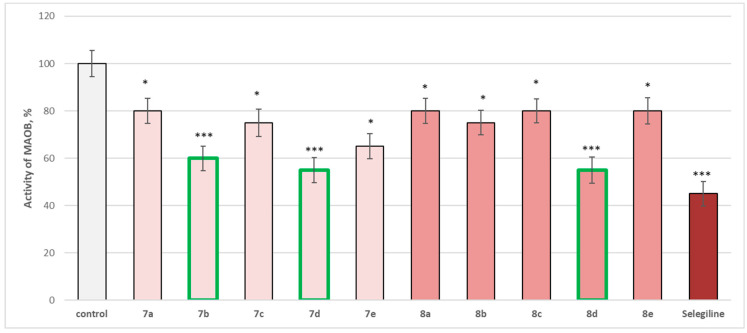
Effect of newly synthetized hydrazones containing a pyrrole cycle in the carboxyl fragment of the structure (at 1 µM concentration) on the activity of the human recombinant MAOB enzyme (*h*MAOB). * *p* < 0.05; *** *p* < 0.001 vs. control (pure *h*MAOB). The green color indicates the most active derivatives.

The entire series was screened for potential MAOA and MAOB inhibitory activity (Figure 14 and Figure 15).

The evaluated target hydrazones, when assessed at 1 µM concentration, did not reveal a statistically significant inhibitory effect on the human recombinant MAOA enzyme (*h*MAOA), compared to the control (pure *h*MAOA) (Figure 14). Only the classical selective MAOA inhibitor clorgilin revealed a statistically significant inhibitory effect by decreasing the enzyme activity by 55% compared to the control (pure *h*MAOA enzyme) (Figure 14).

The whole series of newly synthetized compounds, evaluated at 1 µM concentration, revealed statistically significant inhibitory effect on human recombinant MAOB enzyme (*h*MAOB), with **7a**, **8a**, **8c**, and **8e** inhibiting *h*MAOB with 20%; **7c** and **8b**—inhibiting with 25%; and **7e**—with 35%, compared to the control (pure *h*MAOB) (Figure 15).

The most promising inhibitory effect from the series was observed for compounds **7b**, **7d**, and **8d**, which decreased the enzyme activity as follows: **7b**—with 40%, and **7d** and **8d**—with 45%, compared to the control. The effects of these three compounds were closer to those of selegiline, which inhibited with 55%, compared to the pure *h*MAOB (Figure 15).

The obtained results on the expressed activities against the two isozymes, revealing strong MAOB and a lack of MAOA inhibitory activity, defined our interest in determining the corresponding IC_50_ (EC_50_) of all compounds, aiming to define the molecules with the best *h*MAOB selectivity index (Table 1).

SI: *h*MAOB selectivity index = IC_50_(*h*MAOA)/IC_50_(*h*MAOB) (1)

The obtained values determined compounds **7d** and **8d** as the derivatives with the highest *h*MAOB selectivity with a selectivity index of >204. Thus, these two compounds appeared promising for further research as potential MAOB inhibitors.

### 2.5. Molecular Docking

#### 2.5.1. Molecular Docking in the Active Site of MAOA

The molecular docking simulations in the active site of MAOA (PDB: **2Z5X**) demonstrated that the applied dataset of pyrrole-based compounds did not possess potential inhibitory effect against the former MAO isoform (Table 2).

Glide did not acquire any poses, while GOLD returned insignificant docking scores in MAOA compared to the co-crystallized Harmine. Therefore, the theoretical data suggests that the applied dataset of pyrrole-based compounds are nonactive MAOA inhibitors, thus selective towards MAO type B.

#### 2.5.2. Molecular Docking in the Active Site of MAOB

The molecular docking simulations were carried out with two docking programs—Glide and GOLD 5.3. The consensus docking studies were followed by IFD calculations. The results for MAOB are given in Table 3.

The molecular docking simulations with Glide did not lead to any results, signifying that the searching algorithm in Glide could not return any stable complexes with none of the applied ligands. However, the docking studies with the GOLD genetic algorithm resulted in several applicable scores. All of the ChemPLP fitness scores were lower compared to the co-crystallized MAOB inhibitor—safinamide. The highest docking score was presented by **8b** (137.88), while **8a** achieved a fitness score of 136.49. The standard reversible and selective MAOB inhibitor, safinamide, showed binding energy of −15.20 kcal/mol.

**Figure 16:**
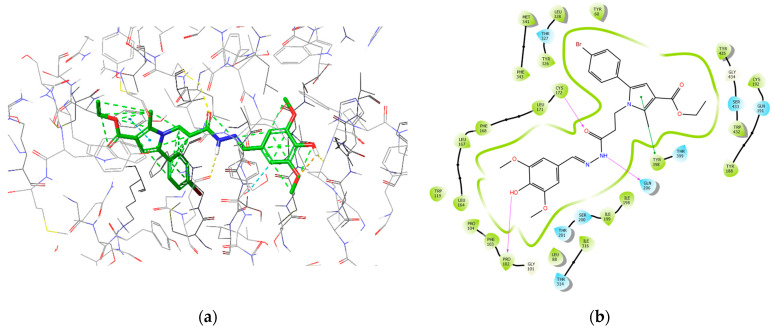
Major intermolecular interactions between the active site of MAOB and **8d**: (**a**) 3D model; (**b**) 2D model.

The most active MAOB inhibitor, 8d, was further examined for its active conformation in the binding site of MAOB (PDB: **2V5Z**). The major intermolecular bonds are provided in Figure 16.

### 2.6. Estimation of BBB Permeability by PAMPA

Compounds **7b**, **7d**, and **8d** were considered the most prospective derivatives in the series based on the MAOB inhibition study. Their ability to penetrate the blood–brain barrier (BBB) was assessed by in silico and in vitro methods as a necessary step in the lead development workflow.

The results from the performed in vitro measurements of the blood–brain barrier penetration, performed by the PAMPA-BBB assay aiming to assess the passive diffusion of the most active structures from the series, are presented in Table 4.

The preliminary PAMPA testing indicated that in relation to the blood–brain barrier (BBB) permeability, the tested pyrrole-based hydrazones may be considered as high permeable, except for 8a and 8e, which were established to be permeable in the medium range with −logP of 5.268 and 5.714, respectively, compared to the applied references.

In addition, a set of physicochemical properties, widely used as criteria for the passage of compounds through BBB, were calculated for the selected compounds [30]. Molecular weight Mw, logP, pKa value, fraction of the ionized molecules f_A_, polar surface area PSA, count of free rotatable bonds FRB, hydrogen bond donors HBD, and hydrogen bond acceptors HBA are given in Table 5, where the fraction of the ionized molecules f_A_ was calculated by the formula:

f_A_ = 10^pH − pKa^/(1 + 10^pH − pKa^, (2)

The computational results show that all tested compounds are relatively lipophilic with logPs from 3.84 to 4.79, but **7b** is moderately polar (PSA 72.69), while **7d** and **8d** possess higher polarity (PSA 111.38). They are very weak acids with pKa between 9.18 and 12.19 and exist as unionized molecules at physiological pH 7.4 as indicated by f_A_.

Thus, keeping in mind these numbers, it is observed that the experimental result correlates well with the in silico studies.

## 3. Discussion

The pathology of Parkinson’s disease (PD) is not fully understood and is complex, with genetic and environmental factors involved. Different hypotheses explain the mechanisms of neurodegeneration, some of them being: Oxidative stress is evaluated in vitro through appropriate animal models of oxidative stress with reagents that mimic oxidative stress, such as 1-methyl-4-phenyl-1,2,3,6-tetrahydropyridine (MPTP), rotenone, 1,1′-dimethyl-4,4′-bipyridinium dichloride (paraquat), and 6-hydroxydopamine (6-OHDA) [31]; mitochondrial dysfunction; protein aggregation [32]; and neuroinflammation [33].

In relation to the current therapeutic strategies, the targeting of monoamine oxidase B (MAOB) inhibitors may contribute to potentially promising therapeutic solutions for Parkinson’s disease.

Although MAOB inhibitors are primarily used in the treatment of PD, dual MAOA/MAOB inhibition is a relatively understudied treatment strategy that may have potential benefits. By inhibiting both isoenzymes, it is possible to reduce the level of several toxic metabolites and to protect different types of neurons, as well as to modulate different neurotransmitter systems. However, the search for new and more effective antiparkinsonian agents continues. Research in this area has concentrated on the development of MAOB inhibitors that are specific and effective but with minimal side effects.

The search for new agents improving and adding to the current symptomatic treatment of neurodegeneration is arising more and more interest. The necessity to decrease the number of animal models used and time spent for evaluation of possible neurotoxic and/or neuroprotective effects leads to the development and application of more cellular and subcellular-based methodologies and in vitro models for assessment.

### 3.1. In Vitro Subcellular Evaluation of the Neurotoxicity Effect of Substances Administered Alone on Biomarkers, Characterizing the Functional-Metabolic Profile of Brain Synaptosomes, Mitochondria, and Microsomes

Attempting to indicate the mechanism of neurological affect, the target compounds were tested on isolated rat brain synaptosomes, mitochondria, and microsomes. The neurotoxicity was determined by evaluation of the cellular viability and the effect of the tested molecules on the corresponding biomarkers affecting the functional-metabolic status of the cells, expressed as a decrease in the reduced glutathione (GSH) levels and an increase in the malondialdehyde (MDA) production.

The applied evaluation of GSH as a biomarker for neurotoxicity was based on the physiological role of this cysteine-containing tripeptide, acting as an antioxidant and cell protector, thanks to its high reactivity to electrophiles. The reactivity assay applied is based on the use of GSH depletion rates to classify the tested molecules into four categories: weak, moderate, strong, and extreme [34]. The appearance of important limitations for these experiments, including the impossibility to account for some permeability aspects such as skin penetration, together with disregarding the possible metabolism transformations and immune recognition, did not decrease their effectivity in distinguishing the target molecules as weak and strong, especially by the further extension towards carbonyl compounds containing (a,b-) unsaturated groups, i.e., aliphatic esters, ketones, and aldehydes [35]. Interestingly, GSH dysfunction is associated with a wide range of pathologies, including neurodegenerative diseases, metabolic diseases such as type II diabetes, chronic immunodeficiencies such as AIDS, anemia, liver diseases, and lung disease, among others. Given the strong association of GSH dysfunction with disease and the diversity of pathological presentation, determining the critical processes impacted by changes in GSH availability remains crucial. [36].

Thus, in the current work, we applied this encouraging toxicological assessment approach and used the GSH assay, aiming to distinguish the compounds affecting the GSH levels from the non-reactive ones.

The obtained results confirmed that the halogen-containing derivatives 7b and 8b may be classified as not reactive to GSH, which may be explained by the fact that their toxicity was consistent with decreased chemical reactivity [37].

Millions of years of evolution have extended GSH’s role as a central reducing agent, and systems developed coupling GSH to critical cell processes including iron homeostasis, electron transport, and reducing power in the form of NADPH. Thus, the GSH/GST antioxidant systems are implicated in the detoxification of xenobiotics, carcinogens, free radicals, and peroxides [38]. The availability of a free hydroxylic group in the structure of the other two least active representatives, **7d** and **8d**, may be considered a logical reason for decreased neurotoxicity.

The low toxicity of the three least toxic molecules is confirmed in all evaluated subcellular structures by the subsequent synaptosomal vitality preservation and low increase in the corresponding MDA levels in all evaluated compartments.

### 3.2. In Vitro Subcellular Evaluation of the Neuroprotective Effect of Target Substances Administered Alone in Different Models of Induced Cellular Toxicity

#### 3.2.1. Effect of Substances in a Model of 6-OHDA-Induced Neurotoxicity in Isolated Rat Brain Synaptosomes

This in vitro model resembles the neurodegenerative processes occurring primarily in PD. The metabolism of 6-OHDA leads to the production of reactive quinones (*p*-quinone), which in turn leads to the formation of ROS. Reactive metabolites and ROS cause damage to the pre- and post-synaptic membrane and lead to neuronal cell damage, which induces dopamine neurotoxicity and neurodegeneration [39].

In this model, the most active were underlined structures **7b**, **7d**, and **8d**. In general, the evaluated derivatives exhibited a neuroprotective effect against the 6-OHDA-induced cellular damage on isolated rat brain cellular and sub-cellular fractions. This was ascertained by synaptosomal viability preservation (Figure 9), preservation of GSH level (Figure 10), and related to this decrease in intracellular ROS production. This suggests the protective potential of the compounds against 6-OHDA-induced oxidative stress.

#### 3.2.2. Effect of Substances in a Model of *t*-BuOOH-Induced Oxidative Stress on Isolated Rat Brain Mitochondria

Another commonly used model substance for evaluation of cellular mechanisms related to oxidative stress-caused cellular alterations is tert-butyl hydroperoxide (*t*-BuOOH). Two possible mechanisms of *t*-BuOOH toxicity have been suggested: depletion of reduced glutathione and oxidation of -SH groups of key mitochondrial enzymes; changes in mitochondrial membrane integrity resulting from the action of free oxygen species that induce a process of lipid peroxidation [40,41,42].

#### 3.2.3. Effect of Substances in a Model of Fe^2+^/AA-Induced Oxidative Stress on Isolated Rat Brain Microsomes

The microsomal fraction obtained by differential centrifugation contains fragments of the endoplasmic reticulum. Microsomes have been used as a model lipid membrane in experiments related to this study of lipid peroxidation processes and the evaluation of the potential antioxidant properties of various biologically active substances [43]. The antioxidant effect of substances is most likely related to their ability to scavenge free radicals.

The neuroprotective effects of the two series **7a**–**7e** and **8a**–**8e**, in different in vitro models of neurotoxicity on brain synaptosomes, mitochondria, and microsomes, are most likely related to their ability: to preserve the level of reduced glutathione (GSH), the major nucleophile scavenger of ROS; to stabilize membranes; and, for the most active, to inhibit MAOB and hence reduce dopamine degradation; this reaction also leads to ROS overproduction.

Four main groups of structural motifs are defined as the most common pharmacophore features contributing to the antioxidative effects of small molecules. They include mainly highly conjugated (e.g., aromatic) hydroxyl groups, amino groups, thiol groups, and isoprenoid groups. Based on this, it is to some extent expected that from all tested hydrazones, the most promising are compounds **7d** and **8d**, containing highly conjugated OH groups. We consider that these molecules operate by the HAT and SET mechanisms.

The results again determined, from most perspectives, the hydrazones **7b**, **7d**, and **8d**. Interestingly, the obtained in vitro results indicated that the derivatives containing a halogen substituent at *p*-position in the phenyl ring found in the side chain or a free OH-group at the same position display better neuroprotective properties.

### 3.3. In Vitro Evaluation of the Enzymatic Activity of the Target Molecules on Human Recombinant MAOA/MAOB Enzyme

Early reports of MAO status in Parkinson’s disease were non-specific for subtypes. The results of measurements of MAOB activity revealed brain protein levels of MAOB as normal or elevated, with MAOB increases generally observed in the levels of astrocyte markers. With regard to MAOA in Parkinson’s disease, the reports of MAOA activities were problematic, variable, and somewhat inconsistent, but the observations revealed surprisingly no decreased concentrations of brain MAOA in Parkinson’s disease. This result defined the current clinical use of MAOB inhibitors (e.g., selegiline, rasagiline) to provide symptomatic benefit in Parkinson’s disease because of the elevation of brain dopamine by inhibiting its breakdown. More speculatively, MAOB inhibitors have been used as potential neuroprotective agents, with the hope that MAOB inhibition might prevent the formation of damaging dopamine-derived oxidation products or the possible conversion of an endogenous/environmental MPTP-like compound to a neurotoxic substance. Although inhibitors of MAOA are used clinically mainly for the treatment of mood disorders, MAOA suppression might also be neuroprotective [44,45,46].

As a result of the extended pharmacological and toxicological studies of the newly synthesized hydrazones containing a pyrrole cycle in the carboxyl fragment of the structure, it was found that the most promising substances for further studies were **7d** and **8d**. These compounds showed the lowest neurotoxicity on subcellular fractions (isolated rat brain synaptosomes, mitochondria, and microsomes). They are also the ones with the most prominent neuroprotective and antioxidant effects in different models of neurotoxicity based on oxidative stress and the formation of reactive metabolites, which in turn lead to ROS overproduction, as in the 6-hydroxydopamine-induced neurotoxicity model in synaptosomes. Of the two series, **7d** and **8d** had the best *h*MAOB selectivity index (>204), making them potential selective MAOB inhibitors.

### 3.4. Molecular Docking

The subsequent IFD calculations demonstrated that **8d** fitted MAOB with the lowest binding energy score (−14.65 kcal/mol), which corresponds to the best solution out of the applied dataset. The latter compound formed three hydrogen bonds with Pro102 (1.84 Å), Cys172 (2.34 Å), and Gln206 (2.18 Å). Compound **8e** showed the second-best score of −14.55 kcal/mol and was involved in two hydrogen bonds with Cys172 (1.97 Å) and Tyr326 (2.32 Å). Moreover, three π–π interactions were formed between **8e** and Tyr326 (5.15 Å), Phe343 (4.22 Å), and Tyr398 (4.18 Å). Importantly, the active amino acids Cys172, Tyr326, and Tyr398 are essential for an enhanced MAOB blocking activity [28].

On the other hand, located at *o*-position in the phenol fragment, two methoxy groups are oriented in the same plane as the aromatic ring, which defines that the 2p-type lone pair of electrons on the oxygen is parallel to the aromatic *p*-orbitals, thereby efficiently overlapping the aromatic π-electron cloud of PhOH [47]. In the case of the presence of two OCH_3_ groups ortho to the phenolic OH (such as in compounds **7d** and **8d**), it is identified that both groups are in the same plane as the phenol ring, with the methyl groups orienting away from the OH [48]. This orientation stabilizes the incipient phenoxy radical (PhO•) and weakens the OH bond of PhOH, facilitating the formation of the additional hydrogen bond.

It appears that the prolongation of the methylene bridge between the central pyrrole core and the side-chain phenyl improves the interaction with the MAOB enzyme and is related to the decreased binding energy related to the better activity results. The obtained molecular docking visualizations identified the appearance of an additional stabilizing hydrogen bond between the free OH group in the phenol-substituent in the structure of **8d** with the Pro102. This type of stabilization is either not available in the other derivatives due to a lack of free OH groups, or in the case of **7d**, the phenolic OH fragment is too far away to interact with the indicated active amino acid.

The hydrazide moiety was located in the substrate pocket, the pyrrole ring together with the ethyl ester group were facing the aromatic cage of MAOB, and the 3,5-dimethoxy-4-hydroxyphenyl fragment was situated in the entrance cavity. Importantly, the top scored after IFD calculations: ligand **8d** formed three stable hydrogen bonds within the active site of MAOB. Pro102 interacted with the *p*-hydroxy group from the 3,5-dimethoxy-4-hydroxyphenyl moiety. The secondary amino group from the hydrazide motif donated a hydrogen to form an H-bond with Gln206, while Cys172 interacted with the carbonyl fragment from the hydrazine group. Interactions with the active amino acids Ile199, Tyr326, Tyr398, and Tyr435 are essential for the MAOB selectivity [49]. Ile199 and Tyr326 and Tyr435 were involved in hydrophobic interactions with 8d. Moreover, Tyr398, which is one of the components of the aromatic cage of MAOB, participated in π–π stabilization with the aromatic pyrrole ring. Thus, selective MAOB inhibitory effects could be observed.

### 3.5. Estimation of BBB Permeability by PAMPA

The design of CNS-acting molecules faces stronger requirements concerning their physicochemical properties. Compound 7b with logP = 4.79, eight free rotatable bonds (FRB), one H-bond donor (HBD), and six H-bond acceptors (HBA) fully meets the requirements for good BBB permeability [50,51,52]. Only the molecular weight of **7b** (Mw 502.79 g/mol) is around the limit of 500 g/mol, proposed as a reasonable threshold for good BBB permeability [53], while the remaining two exceeded it. The remaining two compounds were theoretically predicted as less permeable through BBB, but because of their promising MAOB inhibition potential, they were also subjected to an in vitro PAMPA-BBB assay.

PAMPA provides fast and reliable in vitro methodology to measure the passive diffusion of compounds through an artificial BBB membrane. Since this is the major route for achieving a sufficient drug concentration in the brain, PAMPA ensures a good estimation of the BBB permeation [54]. The results showed that all tested compounds are highly BBB permeable, with −LogPe values of 4.416, 4.819, and 4.464, respectively (Table 4). A correlation is detected between the calculated properties and in vitro permeability of compounds. Lower molecular weight and polarity, higher lipophilicity, and decreased potential of hydrogen bond formation (**7a**) are associated with higher BBB permeability [55].

## 4. Materials and Methods

### 4.1. Chemistry

The synthesis of the pyrrole-based hydrazones evaluated in the manuscript is performed as per the procedure described in Tzankova et al., 2023 [29].

### 4.2. Pharmacological Studies

#### 4.2.1. Chemicals and Reagents

The necessary reagents and buffers for the pharmacological evaluations, including Percoll reagent, Buffer B, glucose, 6-hydroxydopamine (6-OHDA), MTT, dimethyl sulfoxide DMSO, DTNB *tert*-butyl hydroperoxide (*t*-BuOOH), trichloroacetic acid, 0.1 M Tris buffer containing 0.1 mM Dithiothreitol, 0.1 mM Phenylmethylsulfonyl fluoride, 0.2 mM ethylendiamine tetraacetic acid (EDTA), 1.15% potassium chloride (KCl), and 20% (*v*/*v*) glycerol (pH 7.4), ferrous sulfate, ascorbic acid, and the corresponding assay kits for evaluation of MAO inhibitory activity were purchased from Sigma–Aldrich (St. Louis, MO, USA).

#### 4.2.2. Animals

A total of 30 animals were used in the experiments. The animals were obtained from the National Breeding Centre of the Bulgarian Academy of Sciences, Slivnitsa, Bulgaria, and kept under standard conditions in plexiglass cages with free access to water and food and a 12 h/12 h light/dark regime at 20–25 °C. Twelve hours before each specific study, the animals’ food was withdrawn. The experiments were conducted in accordance with Ordinance No. 15 on Minimum Requirements for the Protection and Welfare of Experimental Animals (SG No. 17, 2006) and the European Regulation for the Handling of Experimental Animals. These experiments were also approved from the Bulgarian Food Safety Agency with Permission No. 273, valid till 20 July 2025.

#### 4.2.3. Preparation of Rat Brain Synaptosomes and Mitochondria

Synaptosomes and mitochondria were obtained by multiple, subcellular fractionation using a Percoll gradient [56]. The necessary brain homogenate was prepared and centrifuged at 1000× *g* for 5 min at +4 °C. After centrifugation, the supernatants were collected and centrifuged again at 1000× *g* for 5 min at +4 °C. The supernatants from the two centrifugations were mixed and distributed into four tubes. The tubes were centrifuged at 10,000× *g* for 20 min at +4 °C three times. The last two centrifugations were for purification of synaptosomes and mitochondria.

#### 4.2.4. Isolation of the Corresponding Synaptosomal and Mitochondrial Fractions

The isolation of the required fractions was based on the formation of a colloidal solution of silicon (Percoll), as per the procedure, including: 1. Preparation of a 90% stock solution of Percoll, followed by preparation of two Percoll solutions of different percentages: 16% and 10%. An amount of 4 mL of each of the prepared 16% and 10% Percoll solutions was placed in six test tubes. A 90% Percoll (7.5% Percoll) was added to the precipitate obtained from the previous step from the last centrifugation. The obtained mixtures in all 12 tubes were centrifuged for 20 min at 15,000× *g* +4 °C. After centrifugation, three layers are formed in the tubes. The bottom layer contains mitochondria, the top layer contains lipids, and the middle layer—at the 16% to 10% Percoll limit—contains synaptosomes. The respective layer from each tube is removed through a glass pasteurizer and collected into one. Buffer B + glucose is added to it. The mixture was centrifuged at 10,000× *g* for 20 min at +4 °C. Thus, the isolation buffer is exchanged with the incubation buffer. After centrifugation, the pellet where the synaptosomes are mixed and made up with buffer B + glucose.

#### 4.2.5. Incubation of Synaptosomes

Synaptosomes and mitochondria were incubated with the test substances at a concentration of 50 µM for 1 h.

#### 4.2.6. Establishing and Applying a Dopamine Model of Neurotoxicity

Synaptosomes were incubated with 6-OHDA (150 μM) for 1 h.

#### 4.2.7. MTT Assay to Assess Synaptosomal Viability

After 1 h incubation with the substances and toxic agent, synaptosomes were centrifuged on a microcentrifuge for 1 min at 15,000× *g*. The pellet was mixed gently with buffer B + glucose, and the supernatant where 6-OHDA was discarded to prevent oxidation of MTT. Centrifuge again at 15,000× *g* for 1 min. After the second wash, buffer B + glucose was added to the pellet. To the ‘washed’ synaptosomes, 60 µL of MTT solution is added. The plates were incubated with the MTT solution at 37 °C for 10 min. After incubation, the samples were centrifuged at 15,000× *g* for 2 min. The excess liquid was removed, and a DMSO solution was used to dissolve the formed formazan crystals. After dissolution, the amount of formazan is measured spectrophotometrically at λ = 580 nm [57].

#### 4.2.8. Determination of Reduced Glutathione (GSH) in Isolated Brain Synaptosomes

After precipitation of the proteins with trichloroacetic acid, the thiol groups in the supernatant were determined by DTNB, which produced a yellow-colored compound that absorbs light at λ = 412 nm. After incubation, synaptosomes were centrifuged at 4000× *g* for 3 min. The supernatant was removed and the pellet taken for GSH determination. It was treated with 5% trichloroacetic acid, then left for 10 min on ice. It was centrifuged at 8000× *g* for 10 min (2 °C). The supernatant is taken for GSH determination and frozen at −20 °C. Immediately before measurement, the samples are neutralized with 5N NaOH [58].

#### 4.2.9. Tert-Butyl Hydroperoxide-Induced Oxidative Stress

Isolated brain mitochondria were incubated with 75 µM *tert*-butyl hydroperoxide (*t*-BuOOH) [59].

#### 4.2.10. Determination of Malondialdehyde (MDA) Production in Brain Mitochondria [58]

To the mitochondria was added 0.3 mL of 0.2% thiobarbituric acid and 0.25 mL of sulfuric acid (0.05 M), and the mixture was boiled for 30 min. After boiling, the tubes were placed on ice, and 0.4 mL of n-butanol was added to each, then centrifuged at 3500× *g* for 10 min. The amount of MDA was determined spectrophotometrically at 532 nm.

#### 4.2.11. Determination of GSH Level in Brain Mitochondria [60]

After incubating the mitochondria with the substances and tert-butyl hydroperoxide, the reaction was stopped with 5% trichloroacetic acid, and each sample was homogenized with the acid and left on ice. After centrifugation of the homogenate at 6000× *g*, a 0.04% solution of DTNB was added to the supernatant to give a yellow color, the determination being spectrophotometric at 412 nm.

#### 4.2.12. Isolation of Brain Microsomes [61]

The brain was homogenized in 9 volume parts of 0.1 M Tris buffer containing: 0.1 mM dithiothreitol, 0.1 mM phenylmethylsulfonyl fluoride, 0.2 mM EDTA, 1.15% KCl, and 20% (*v*/*v*) glycerol (pH 7.4). The resulting homogenate was centrifuged twice at 17,000× *g* for 30 min. The supernatants from the two centrifugations were pooled and centrifuged twice at 100,000× *g* for 1 h. The pellet was frozen in 0.1 M Tris buffer.

#### 4.2.13. Iron/Ascorbate-Induced Lipid Peroxidation (LPO) 

Non-enzyme-induced lipid peroxidation was induced with 20 μM ferrous sulfate solution and 0.5 mM ascorbic acid solution [62]. 

#### 4.2.14. Determination of MDA in Brain Microsomes [62]

After completion of incubation of the microsomes with the substances and toxic agent, the reaction was stopped by the addition of 0.5 mL of 20% trichloroacetic acid, followed by 0.5 mL of 0.67% thiobarbituric acid. The ongoing reactions are associated with the formation of a colored complex between the malondialdehyde formed and thiobarbituric acid. The determination of MDA was spectrophotometric at 535 nm. A molar extinction coefficient of 1.56 × 105 M^−1^ cm^−1^ was used for the calculation.

#### 4.2.15. Determination of Human Recombinant MAOA/B Enzyme Activity

The activity of recombinant human MAOA/B was determined fluorometrically. Tyramine hydrochloride was used as substrate. The activity is determined by detection of H_2_O_2_ production. This production is in turn reported by binding to horseradish peroxidase using *N*-acetyl-3,7-dihydroxyphenoxazine (AmplexRed, Thermo Fisher Scientific, Waltham, MA, USA) [63].

Working solutions of the test substances, reagents, and human recombinant MAOA/B enzyme (*h*MAOA/B) were prepared in reaction buffer according to the manufacturer’s instructions. A pure working solution of MAOA/B in reaction buffer, a working solution of MAOA/B containing hydrogen peroxide, and a pure reaction buffer were used as controls. Substances were applied at a final concentration of 1 µM. The substances together with *h*MAOB were placed in a 96-well plate (8 samples for each substance), and then the plate was placed in an incubator for 30 min (in the dark, at 37 °C). Fluorimetric readings were performed in a Synergy 2 Microplate Reader at two wavelengths (570 nm and 690 nm).

#### 4.2.16. Statistical Methods

The results of the experiments performed on isolated brain synaptosomes, mitochondria, and microsomes were statistically processed using the ‘MEDCALC’ program using the non-parametric Mann–Whitney method at significance levels *p* < 0.05, *p* < 0.01, and *p* < 0.001.

The results obtained from *h*MAOA/B activity were statistically processed using GraphPad Prism 5.0 software.

### 4.3. Molecular Docking

For the purpose of this study, the crystallographic structures of *h*MAOB (ID: 2V5Z) and MAO-A (ID: 2Z5X) were retrieved from the Protein Data Bank (PDB). Subsequently, the proteins were prepared by the Protein Preparation Wizard workflow available in Maestro (Schrödinger Release 2022-1: Protein Preparation Wizard; Epik, Schrödinger, LLC, New York, NY, USA, 2021; Impact, Schrödinger, LLC, New York, NY, USA; Prime, Schrödinger, LLC, New York, NY, USA, 2021). The workflow incorporated the generation of hydrogen atoms and het states, assignment of bond orders, and the removal of non-active water molecules. The active waters were retained and incorporated in the docking studies. The amino acids were ionized at a physiological pH. The final protein was minimized with the OPLS4 force field. The title ligands were drawn with 2DSketcher in Maestro. The Ligprep module of Maestro (Schrödinger) was utilized for the ligand’s preparation. Hydrogen atoms were added, and ionization states at physiological pH were generated. Finally, the geometries were minimized with the OPLS4 force field. The docking simulations were carried out with both GOLD 5.3 and Glide (Schrödinger). The grid boxes were generated around each co-crystallized ligand—safindamide for 2V5Z and Harmine for 2Z5X. To explore the role of the active amino acids, induced-fit docking (IFD) simulations were introduced. The IFD considers the side chains as fully flexible. All calculations were carried out on an AMD Ryzen 9 5950X 16 core CPU and 64 GB of installed RAM. The operating system was Windows 10 Pro.

### 4.4. PAMPA-BBB Assay

In vitro parallel artificial permeability assay—blood–brain barrier (PAMPA-BBB) test was applied by using PAMPA Permeability Analyzer (Pion Inc., Billerica, MA, USA). The effective permeabilities (Pe) of compounds were measured following the manufacturer’s BBB Protocol. Briefly, 10 mM stock solutions of the evaluated and reference compounds in DMSO were diluted with Prisma HT buffer pH 7.4 (Pion Inc.) to achieve a final concentration of 50μM, and the donor plate of PAMPA “sandwich” was loaded with 200 µL samples. The membranes of the acceptor plate were impregnated with BBB-1 lipid (Pion Inc.), and the wells were filled with 200 µL BSB (Brain Skin Buffer, Pion Inc.). The plates were assembled with the acceptor compartment above the donor one. Incubation was set up for 4 h at 25 °C with no stirring in the Gut-Box. To assess the permeability of compounds, their concentrations in both the donor and acceptor phases were spectrophotometrically determined (250–500 nm) after the end of incubation. Blank and reference samples were also UV-scanned at the same wavelength range. Effective permeability coefficients Pe (10^−6^ cm/s) were calculated by PAMPA Explorer Command Software (Ver 3.8) as an average of three replicates and presented as −logPe. A compound was predicted as highly permeable if −logPe < 5 and low permeable if −logPe > 6. When 6 < −logPe < 5, the compound was classified as medium permeable [64]. Theophylline, corticosterone, and propranolol. HCl were used as reference standards for low, medium, and high permeability, respectively [65].

### 4.5. Physicochemical Properties Evaluation of the Molecules

Molecular weight Mw and logP for the target compounds were calculated by the SwissADME tool [66]. The corresponding pKa values, polar surface area (PSA), count of free rotatable bonds (FRB), hydrogen bond donors (HBD) and hydrogen bond acceptors HBA, and factor of ionization (f_A_) were calculated by ACD/logD software v. 9.08, ACD Inc., Toronto, ON, Canada.

## 5. Conclusions

The extended pharmacological and toxicological studies on subcellular fractions (isolated rat brain synaptosomes, mitochondria, and microsomes) for series 7a-7e and 8a-8e revealed that when administered alone, at a concentration of 50 µM, all substances exhibited weak neurotoxic effects relative to the control (untreated subcellular fractions), with **7b**, **7d**, and **8d** defined as the least toxic. The most prominent neuroprotective antioxidant effects of the two series, in different models of neurotoxicity based on overproduction of ROS and induction of oxidative stress, were determined for compounds **7b**, **7d**, and **8d**.

In addition, the corresponding inhibitory activity towards human MAOA and MAOB isozymes was evaluated, revealing a lack of MAOA effects and good MAOB activity at 1 µM concentration. Two of the evaluated molecules, **7d** and **8d**, showed the best *h*MAOB selectivity index (>204), making them potential selective MAOB inhibitors.

The appearance of selective MAOB inhibitory effects is based on the interaction of the tested molecules with Tyr398, which is one of the components of the aromatic cage of MAOB and participated in π–π stabilization with the aromatic pyrrole ring. The preliminary PAMPA testing indicated that in relation to the blood–brain barrier (BBB) permeability, the tested pyrrole-based hydrazones may be considered as high permeable, except for **8a** and **8e**, which were established to be permeable in the medium range with −logP of 5.268 and 5.714, respectively, compared to the applied references.

## Figures and Tables

**Table 1:** IC_50_ (EC_50_) of whole series newly synthetized hydrazones containing a pyrrole cycle in the carboxyl fragment of the structure, selegiline, clorgilin on *h*MAOA/B.

Compounds IDs	IC_50_ (EC_50_), (µM ± SD) *h*MAOA	IC_50_ (EC_50_), (µM ± SD) *h*MAOB	SI
**7a**	>100	0.812 ± 0.10	>123
**7b**	>100	0.551 ± 0.10	>181
**7c**	>100	0.815 ± 0.20	>123
**7d**	>100	0.491 ± 0.09	>204
**7e**	>100	0.810 ± 0.09	>123
**8a**	>100	0.821 ± 0.10	>122
**8b**	>100	0.815 ± 0.10	>123
**8c**	>100	0.814 ± 0.20	>123
**8d**	>100	0.490 ± 0.09	>204
**8e**	>100	0.817 ± 0.09	>122
Selegiline	-	0.320 ± 0.20	
Clorgilin	18.74 ± 0.096	-	>123

**Table 2:** Molecular docking simulations in MAOA (PDB: **2Z5X**).

Compound	Glide kcal/mol	ChemPLP	IFD kcal/mol
**7a**	n.a	39.81	n.a
**7b**	n.a	50.47	n.a
**7c**	n.a	22.19	n.a
**7d**	n.a	13.58	n.a
**7e**	n.a	33.67	n.a
**8a**	n.a	36.54	n.a
**8b**	n.a	43.09	n.a
**8c**	n.a	35.57	n.a
**8d**	n.a	0.68	n.a
**8e**	n.a	29.85	n.a
Harmine	−6.05	78.50	−9.53

n.a—not available.

**Table 3:** Molecular docking results in MAOB (PDB: **2V5Z**).

Compound	Glide kcal/mol	ChemPLP Fitness Score	IFD kcal/mol	Intermolecular Stabilization
**7a**	n.a	126.52	−13.80	H-bonds (Tyr188; Tyr435); π–π (Tyr326)
**7b**	n.a	121.99	−13.71	H-bonds (Cys172; Ile198); π–π (Tyr326; Tyr435
**7c**	n.a	117.94	−13.83	H-bond (Gln206); π–π (Tyr60; Tyr398)
**7d**	n.a	103.00	−14.45	H-bond (Tyr435)
**7e**	n.a	115.90	−13.66	Halogen bond (Trp119)
**8a**	n.a	136.49	−13.16	π–π (Trp119)
**8b**	n.a	137.88	−14.27	H-bond (Cys172); π–π (Phe343; Tyr398)
**8c**	n.a	133.28	−13.73	H-bond (Cys172); π–π (Phe343)
**8d**	n.a	121.03	−14.65	H-bonds (Pro102; Cys172 Gln206); π–π (Tyr398)
**8e**	n.a	123.95	−14.55	H-bonds (Cys172; Tyr326); π–π (Tyr326; Phe343; Tyr398)
** Safinamide	−12.19	168.27	−15.20	H-bonds (Gln206; H_2_O)

n.a—not available; ** Co-crystallized, selective MAOB ligand.

**Table 4:** Results from the performed in vitro measurements of the blood–brain barrier penetration for the evaluated compounds **7a**–**7e**, **8a**–**8e**.

PAMPA ID	Mw g/mol	PAMPA BBB −logPe
**7**	380.00	4.864 ± 0.001
**7a**	511.42	4.849 ± 0.043
**7b**	502.80	4.416 ± 0.022
**7c**	513.35	4.601 ± 0.040
**7d**	544.40	4.819 ± 0.004
**7e**	528.40	4.814 ± 0.15
**8**	393.28	4.538 ± 0.001
**8a**	525.45	5.268 ± 0.014
**8b**	516.82	4.444 ± 0.004
**8c**	527.37	4.631 ± 0.047
**8d**	558.43	4.464 ± 0.026
**8e**	542.43	5.714 ± 0.041
Theophylline	**180.167**	6.553 ± 0.025 low
Corticosterone	**346.467**	5.202 ± 0.020 medium
Propranolol HCl	**259.349**	4.389 ± 0.014 high

**Table 5:** Experimentally measured BBB permeability by PAMPA assay and in silico calculated physicochemical properties of compounds selected for PAMPA analysis: Mw—molecular weight, logP, pKa value, f_A_—fraction of the ionized molecules, PSA—polar surface area; FRB—free rotatable bonds; HBD—hydrogen bond donors; HBA—hydrogen bond acceptors.

ID	PAMPA BBB −logPe	MW g/mol	logP	pKa	f_A_	PSA, Å^2^	FRB	HBD	HBA
**7b**	4.416	502.79	4.79	10.64	0.00	72.69	8	1	6
**7d**	4.819	544.39	3.84	9.18	0.02	111.38	11	2	9
**8d**	4.464	558.42	4.04	12.19	0.00	111.38	12	2	9

## Data Availability

Data are contained within the article.
